# Targeting TLR4 with ApTOLL Improves Heart Function in Response to Coronary Ischemia Reperfusion in Pigs Undergoing Acute Myocardial Infarction

**DOI:** 10.3390/biom10081167

**Published:** 2020-08-09

**Authors:** Rafael Ramirez-Carracedo, Laura Tesoro, Ignacio Hernandez, Javier Diez-Mata, David Piñeiro, Macarena Hernandez-Jimenez, Jose Luis Zamorano, Carlos Zaragoza

**Affiliations:** 1 Cardiology Department, Universidad Francisco de Vitoria/Hospital Ramón y Cajal Research Unit (IRYCIS), CIBERCV, 28223 Madrid, Spain; rrcarracedo@hotmail.com (R.R.-C.); lauratesoro4@hotmail.com (L.T.); naxete1992@gmail.com (I.H.); jdiezmata@gmail.com (J.D.-M.); 2 Aptatargets SL, Avda, Cardenal Herrera Oria 298, 28035 Madrid, Spain; d.pineiro@aptatargets.com (D.P.); m.hernandez@aptatargets.com (M.H.-J.); 3 Cardiology Department, IRYCIS, CIBERCV, 28034 Madrid, Spain; zamorano@secardiologia.es

**Keywords:** toll-like receptor 4, ApTOLL, acute myocardial infarction, cytokines, matrix metalloproteinases

## Abstract

Toll-like receptor 4 (TLR4) contributes to the pathogenesis of coronary ischemia/reperfusion (IR). To test whether the new TLR4 antagonist, ApTOLL, may prevent coronary IR damage, we administered 0.078 mg/kg ApTOLL or Placebo in pigs subjected to IR, analyzing the levels of cardiac troponins, matrix metalloproteinases, pro-, and anti-inflammatory cytokines, heart function, and tissue integrity over a period of 7 days after IR. Our results show that ApTOLL reduced cardiac troponin-1 24 h after administration, improving heart function, as detected by a significant recovery of the left ventricle ejection fraction (LVEF) and the shortening fraction (FS) cardiac parameters. The extension of necrotic and fibrotic areas was also reduced, as detected by Evans blue/2,3,5-triphenyltetrazolium chloride (TTC) staining, Hematoxylin/Eosine, and Masson Trichrome staining of heart sections, together with a significant reduction in the expression of the extracellular matrix-degrading, matrix metalloproteinase 9. Finally, the expression of the following cytokines, CCL1, CCL2, MIP1-A-B, CCL5, CD40L, C5/C5A, CXCL1, CXCL10, CXCL11, CXCL12, G-CSF, GM-CSF, ICAM-1, INF-g, IL1-a, ILI-b, IL-1Ra, IL2, IL4, IL5, IL6, IL8, IL10, IL12, IL13, IL16, IL17-A, IL17- E, IL18, IL21, IL27, IL32, MIF, SERPIN-E1, TNF-a, and TREM-1, were also assayed, detecting a pronounced decrease of pro-inflammatory cytokines after 7 days of treatment with ApTOLL. Altogether, our results show that ApTOLL is a promising new tool for the treatment of acute myocardial infarction (AMI).

## 1. Introduction

Acute myocardial infarction (AMI) is a major cause of morbidity and mortality worldwide [1]. Following coronary occlusion, ischemia leads to cardiac cell death [2], and, depending on the necrotic extension, an adverse remodeling can worsen recovery, leading to chronic heart failure (CHF), and hence impair the patient′s outcome [2].

Cardiac remodeling is a key step that will determine the severity of heart failure. It all starts in response to an injury sensed by an inflammatory response, which, in the case of AMI, is mediated by infiltration and activation of several cell types at the foci of inflammation, with the ultimate goal of removing dead cells and tissue repair. The process ends with the inflammation resolution phase, in which resolutive immune cells and fibroblasts promote cell proliferation, angiogenesis, and a replenishment of dead tissue with a collagen-based scar [3,4].

Among the different sensors of inflammation, toll-like receptors (TLRs) represent a key molecular link between innate and adaptative responses, mediating tissue injury and inflammation. Toll-like receptor 4 (TLR4) is a transmembrane signaling protein, which is the major pattern recognition receptor expressed by several cell types. Besides bacterial lipopolysaccharide, TLR4 recognizes a variety of ligands, which are released in response to myocardial ischemia/reperfusion, resulting in the activation of TLR4 inflammatory signaling pathway in the heart, as a mechanism to increase the expression and nuclear translocation of nuclear factor kappa-B (NF-kappa-B) transcription factor [5], which leads to the expression of pro-inflammatory cytokines, including TNF-α, interleukin-1β and interleukin-6, chemokines, the recruitment of lymphocytes, increasing the expression of inducible nitric oxide synthase and matrix degrading enzymes [6], and leading to the acute-inflammatory phase and a source of cardiac cell necrosis. While inflammation is a necessary element during the early stage of remodeling, a prolonged inflammatory response may impair cardiac physiology by promoting left ventricle dilation and excessive scar formation [7]. Hence, timely control of the inflammatory response is crucial to prevent the severity of HF.

Several groups have developed different strategies in mice to study the contribution of TLR4 activation in the cardiac function in response to cardiac ischemia/reperfusion (IR) [8,9,10], and while all the studies agree on the effect on cardiac necrosis, a disparity of results arise when studying the effect of TLR4 on cardiac function.

Small non-coding RNAS, including RNA aptamers, have been tested in experimental diagnostic and therapeutic diseases [11], including myocardial infarction [12]. ApTOLL is a new therapeutic tool designed to antagonize TLR4. ApTOLL aptamer is a single-stranded oligonucleotide that recognizes and specifically binds to TLR4 by structural interaction, as described [13]. Once folded under physiological conditions, aptamers acquire unique three-dimensional structures based on their nucleotide sequence, conferring target affinity and specificity [14,15,16]. The protective effect of ApTOLL was recently reported against experimental strokes, a setting in which TLR4 played a crucial role in the initiation of ischemic injury [13].

The present study aimed to evaluate the potential therapeutic effect of ApTOLL as a cardioprotective compound in a porcine model of cardiac IR.

## 2. Materials and Methods 

### 2.1. Reagents and Equipment

Hematoxylin-eosin (H/E), Trichrome Masson staining reagents, TTC, Evans blue, and fetal bovine serum were from Sigma (Madrid, Spain). Horseradish peroxidase (HRP)-conjugated anti-mouse secondary antibody and liquid 3,3′-diaminobenzidine (DAB) substrate were from Dako (Santa Clara, CA, USA). The anti-Matrix Metalloprotease 9 (MMP-9) antibody and Human Cardiac Troponin 1 Simple-Step ELISA Kit (ab200016) and the Tunel assay kit (ab206386) were from Abcam (Cambridge, UK); the proteome Profiler Array (ARY005B) was from R&D Systems (Minneapolis, MN, USA); the ketamine was from Pfizer (New York, NY, USA); the isoflurane was from Abbvie (North Chicago, IL, USA); the propofol was from Fresenius (Bad Homburg, Germany); the fentanyl was from Kern Pharma (Madrid, Spain); the diazepam was from Roche (Basel, Switzerland); and the amiodarone was from Sanofi Aventis (Gentilly, France).

The following is a list of the most common equipment used for this investigation: The 5415R Refrigerated Centrifuge was from Eppendorf (Hamburg, Germany). The chemiluminescence imaging system Fusion Solo-S and the image analysis software Fusion-Capt were from Vilber-Lourmat (Eberhardzell, Germany). The TCS-SP5 Confocal Microscope was from Leica (Wetzlar, Germany). The microplate reader was from Biotek (Winooski, VT, USA). The NanoDrop One Spectrophotometer was from Thermo Scientific (Waltham, MA, USA). Guiding catheters, angioplasty balloons, and catheter introducers were from Cordis (Miami, FL, USA). Diagnostic and steerable guidewires were from Boston Scientifics (Malborough, MA, USA). The balloon inflation devices and midazolam were from B.Braun (Melsungen, Germany).

### 2.2. Animal Model of Coronary Ischemia/Reperfusion

Animal procedures were performed in the Experimental Surgery Department of the Hospital Universitario La Paz (Madrid, Spain), as described [17,18]. The investigation conformed to the Guide for the Care and Use of Laboratory Animals, published by the US National Institutes of Health (NIH Publication No. 85-23, revised 1985) and the Animal Welfare Ethics Committee and complied with the EU Directive on experimental animals (63/2010 EU) and related Spanish legislation (RD 53/2013). PROEX 365-15.

Before experimentation, animals were housed for one week to avoid cardiovascular responses derived from anxiety associated with the new environment. Prior to experimentation, the animals underwent a cardiac ultrasound to check for abnormalities of cardiac anatomy and function (Figure S1). 

Yorkshire female pigs (37.8 ± 5.2 kg) were pre-medicated with intramuscular ketamine 10 mg/kg (Pfizer) and midazolam 0.5 mg/kg (B.Braun). Anesthesia was induced by inhaled isoflurane (Abbvie) and maintained with continuous infusion of propofol 2mL/kg/h (Fresenius), fentanyl 50 µg/kg/h (Kern Pharma), and diazepam 10 µg/kg/h (Roche). Animals were intubated and ventilated with 100% oxygen saturation. The animals received 5000 IU of heparin and amiodarone 2 mg/kg/h (Sanofi Aventis) to avoid blood clotting of catheters and malignant cardiac arrhythmias, respectively. Before balloon inflation, coronary anatomy was visualized to check for vasculature abnormalities.

Ischemia/reperfusion was induced by LAD occlusion for 45 min, using a JL 3 6F catheter and an angioplasty balloon (inflated to the pressure of eight atmospheres). In cases when ventricular fibrillation/ventricular tachycardia occurred, we administered a biphasic DC shock (10–20 joules), combined with direct manual chest compressions. After 45 min of LAD occlusion, the artery was unblocked, and after 10 min of reperfusion, APTOLL was administered intravenously in slow bolus at a dose of 0.078 mg/kg (equivalent dosage previously tested in mice [13]) and diluted in distilled water.

The animals were then randomized to a control group (n = 10) or treated with APTOLL (n = 10). A total of 10 mL of arterial blood was obtained from the femoral artery at the following times: before AMI, 50 min, 75 min, 2 h, 8 h, 24 h, 3 days, and 7 days post-AMI. Immediately after collection, plasma was isolated by centrifugation at 3000 rpm for 10 min and all the samples were kept at −80 °C before experimentation and stored for future investigation. 

Overall, 20% of pigs that were treated with a placebo and 10% that were treated with APTOLL died by day 1 after treatment.

### 2.3. Echocardiography

Pig hearts were visualized by echocardiography by using a Vivid Q ultrasound system from GE healthcare (General Electric, Chicago, IL, USA), equipped with a 1.9-4 MHz scan head. In anesthetized animals, parasternal short-axis-view images of the heart were recorded in a B-mode to allow M-mode recordings by positioning the cursor in the parasternal short-axis view perpendicular to the interventricular septum and posterior wall of the left ventricle. From these recordings, the following parameters were determined using the on-site software cardiac package: systolic and diastolic interventricular septum thickness (IVS), systolic and diastolic left-ventricle internal diameter (LVID), systolic and diastolic left-ventricle posterior wall thickness (LVPW), left-ventricle ejection fraction (EF), left ventricle shortening fraction (FS), heart rate (HR), and cardiac output (CO). In addition, left ventricle ejection fraction (LVEF) was also measured by using the B-mode Simpson biplane method, using 4 chamber and a 2 chamber left ventricle long axis views, with similar results. To avoid inter-observer derived biases, data acquisition and analysis was performed by one single operator.

### 2.4. Evans Blue/TTC Staining

The extension of myocardial infarction was evaluated by Evans blue perfusion and TTC staining. By day 7, a catheter inflated at the same position, as in day 0, to avoid Evans blue perfusion downstream to the area at risk, and a pigtail catheter was inserted from the femoral artery and placed up to the left ventricle for Evans blue perfusion into the systemic circulation. After 1 min of perfusion, the animals were sacrificed by injection of a potassium chloride solution, and the hearts were then isolated, washed three times with saline buffer, frozen for 12 h at –20 °C, and chopped into 0.5 cm slices from base to apex. The slices were incubated with 1% TTC dye, dissolved in a saline buffer for 20 min at 37 °C, and then washed for 20 min with 10% paraformaldehyde. Images were acquired with the ImageJ software, discriminating between healthy areas (blue) from the area at risk (dark red) and the pale necrotic area (white), calculating the area of necrosis as percentage respect to the area at risk.

### 2.5. Confocal Microscopy

Paraffin embedded 0.5 µm heart sections were incubated with anti-MMP-9 (diluted 1:500 in PBS 1.5% BSA) primary antibody overnight at 4 °C. After washing three times with PBS, the slides were incubated with Alexa-Fluor-647 conjugated secondary antibody for 1 h at room temperature. Slides were washed three times with PBS and mounted in PBS media, containing Hoechst for nuclei visualization. Images were taken using a Leica TCS SP5 confocal microscope. At least three different fields per condition were obtained.

### 2.6. Determination of Cardiac TroponinI

Plasma Troponin I was determined with the commercial Human Cardiac Troponin 1 SimpleStep ELISA Kit (Abcam), following the manufacturer’s instructions.

### 2.7. Histology and Immunohistochemistry

Histological, immunohistochemical, and immunohistofluorescence procedures were performed, as previously described [19,20].

### 2.8. Statistical Analysis

All values were given as mean ± S.D. Significance i reported at the 5% level. Whenever comparisons were made with a common control, significance of differences was tested by Dunnett′s modification of the t-test.

## 3. Results

### 3.1. ApTOLL Induces Cardiac Protection in Response to IR 

**Figure 1:**
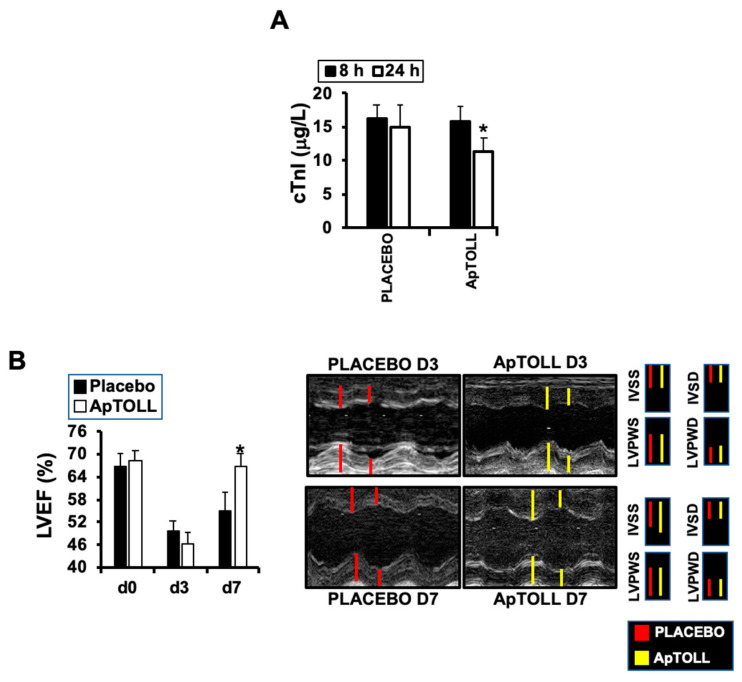
(**A**) Cardiac Troponin I (cTnI) levels in plasma collected from pigs treated with 0.078 mg/kg ApTOLL or Placebo at 8 and 24 h after ischemia/reperfusion (IR) (N = 10/group, mean ± SD * *p* < 0.002 ApTOLL). (**B**) Left ventricle ejection fraction levels detected by cardiac ultrasound at the times indicated. Representative M-Mode images from Placebo and ApTOLL treated animals, in which end diastolic and systolic thickness of the interventricular septum thickness (IVS) and left-ventricle posterior wall thickness (LVPW) are represented (N = 10/group (d0), N = 9 ApTOLL/8 Placebo (d3, and d7). Mean ± SD * *p* < 0.001 ApTOLL vs. Placebo).

**Figure 2:**
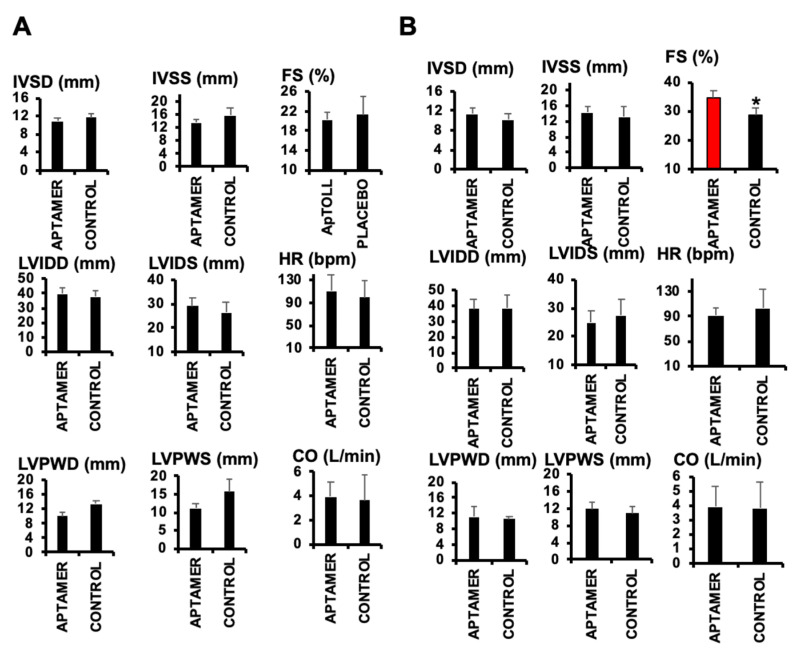
Echocardiographic parameters collected by day 3 (**A**) and day 7 (**B**), respectively, after IR. (N = 9 ApTOLL/8 Placebo. Mean ± SD. EF: * *p* < 0.0006 ApTOLL vs. Placebo. FS: * *p* < 0.003 ApTOLL vs. Placebo).

To test the effect of ApTOLL, we first determined cardiac troponin-I levels, indicative of ischemic injury [21] at the times indicated, detecting a significant reduction 24 h after IR in pigs treated with 0.078 mg/kg ApTOLL vs. Placebo (Figure 1A). Cardiac ultrasound also revealed a significant reduction of the left ventricle ejection fraction (LVEF) by day 7 in the same pigs (Figure 1B). The rest of the parameters examined did not show differences, with the exception of the shortening fraction (FS), which was reduced by day 7 after treatment with ApTOLL (Figure 2, Table 1).

### 3.2. ApTOLL Reduces Left Ventricle Necrosis and Fibrosis by Day 7 after IR

**Figure 3:**
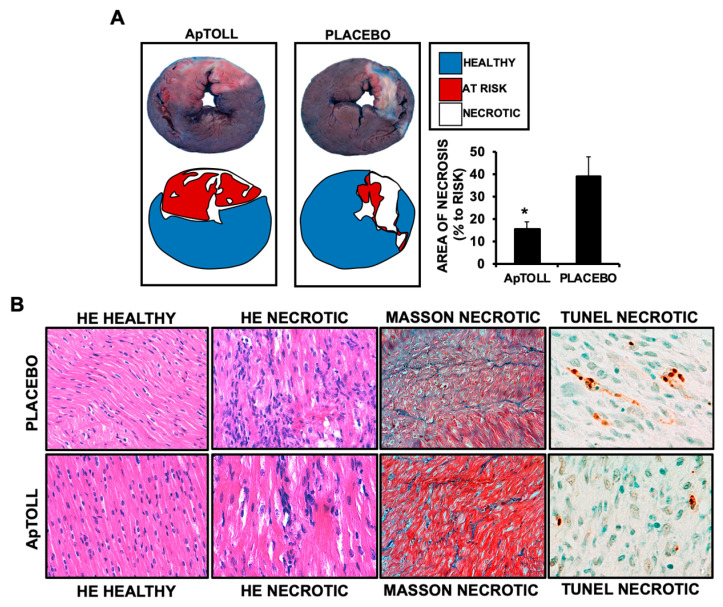
(**A**) Evans blue/TTC double staining performed in 0.5 cm heart sections isolated 7 days after IR from pigs treated with 0.078 mg/kg ApTOLL of Placebo (N = 9 ApTOLL/8 Placebo. Mean ± SD. * *p* < 0.002 Placebo (Vehicle) vs. ApTOLL). (**B**) Left panels: H/E staining of heart sections from healthy and necrotic areas of hearts collected from pigs treated with 0.078 mg/kg ApTOLL or Placebo. Middle Right panel: Masson Trichrome staining of necrotic sections. Right panel. Tunel assay staining of necrotic sections. N = 5 ApTOLL/4Placebo.

We next asked whether ApTOLL-induced improvement of cardiac function was due to preventing cardiac necrosis. Evans blue/TTC double staining (see methods for details) showed that after 7 days of treatment, the necrotic area was significantly reduced in the hearts of pigs treated with ApTOLL (Figure 3A). In the same way, H/E, Masson Trichrome, and Tunel staining of ventricular sections showed reduced fibrosis, necrosis, and apoptosis in response to ApTOLL (Figure 3B), suggesting the antagonizing of TLR4, leading to inhibition of the downstream necrotic molecular cascade.

### 3.3. APTOLL Inhibits the Expression of MMP-9 by Day 7 after IR

**Figure 4:**
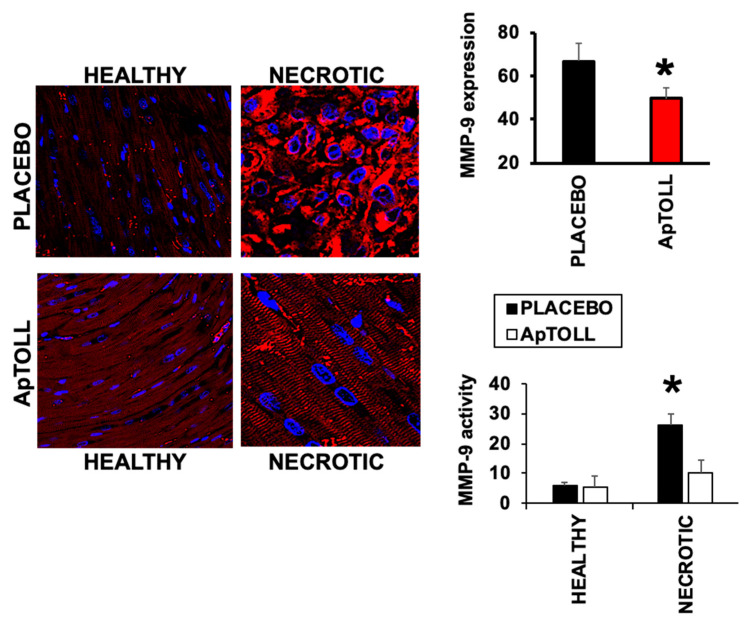
Confocal microscopy detection of Matrix Metalloprotease 9 (MMP-9; red) in heart sections of pigs treated with 0.078 mg/kg ApTOLL or Placebo, 7 days after IR. Nuclei were stained with DAPI (N = 5 ApTOLL/4 Placebo. Mean ± SD. * *p* < 0.001 Placebo vs. ApTOLL). Lower graph. MMP-9 activity assay in the same hearts (N = 3/condition. Mean ± SD. * *p* < 0.004 Necrotic Placebo vs. Necrotic ApTOLL).

Degradation of the extracellular matrix is a critical step during myocardial remodeling and repair. Matrix Metalloprotease 9 (MMP-9), a marker of adverse remodeling [22], plays a key role in this process as it degrades the extracellular matrix components. In pigs treated with ApTOLL, the expression of MMP-9 by day 7 after IR was significantly reduced by 40%, compared to the Placebo group, as detected in the same heart sections as before by confocal microscopy immunofluorescence, with specific anti-MMP-9 antibodies. In the same way, MMP-9 activity assay also revealed a significant reduction in response to ApTOLL by day 7 after treatment (Figure 4).

### 3.4. ApTOLL Reduces the Expression of Pro-Inflammatory Cytokines 7 Days After Reperfusion

To test the effect of antagonizing TLR4, the level of 36 cytokines and chemokines was assessed in plasma collected 7 days after IR. We evaluated the levels of CCL1, CCL2, MIP1-A-B, CCL5, CD40L, C5/C5A, CXCL1, CXCL10, CXCL11, CXCL12, G-CSF, GM-CSF, ICAM-1, INFG, IL1-A, ILI-B, IL-1RA, IL2, IL4, IL5, IL6, IL8, IL10, IL12, IL13, IL16, IL17-A, IL17- E, IL18, IL21, IL27, IL32, MIF, SERPIN-E1, TNF-A, and TREM-1, detecting significant differences in 24 cytokines (Table 2).

**Figure 5:**
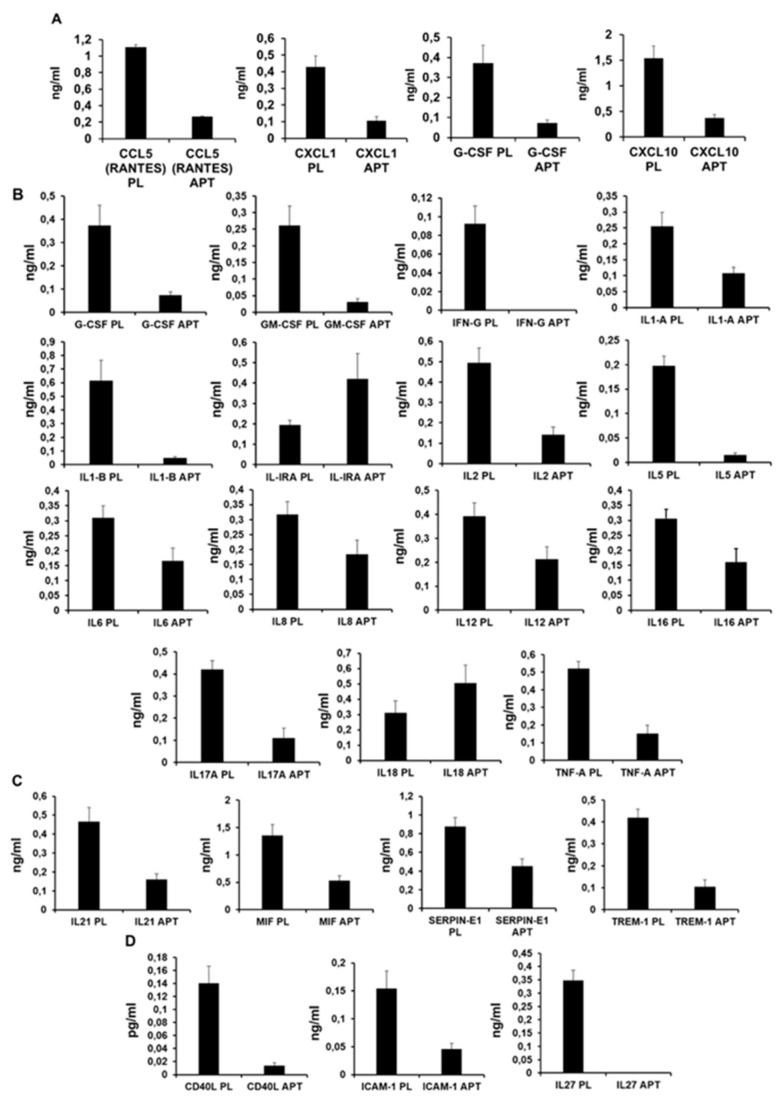
Plasma levels of selected cytokines detected by day 7 after IR. (**A**) Chemokines; (**B**) pro-inflammatory cytokines; (**C**) resolving cytokines; (**D**) miscellaneous. All cytokines depicted presented statistically significant differences between ApTOLL vs. PLACEBO, with *p* values < 0.001. (N = 9 ApTOLL/8 PLACEBO. Mean ± SD).

In particular, ApTOLL induced chemokine downregulation of CCL5, CXCL1, G-CSF, CXCL10 (Figure 5A), and pro-inflammatory cytokines, GM-CSF, IFN-γ, IL-1α, IL-1β, IL-2, IL5, IL6, IL-8, IL-12, IL-16, IL-17A, IL-18, and TNFα. It is important to highlight IL-1, IL-6, IL-8, and TNF-α for their key roles in the inflammatory response during an AMI. However, the levels of IL-18 were upregulated in response to ApTOLL (Figure 5B). ApTOLL also upregulated the levels of IL-1RA (Figure 5C or D), an anti-inflammatory cytokine, but downregulated the levels of resolving cytokines IL-21, MIF, SERPIN-E1, and TREM-1 (Figure 5C, together with CD40, ICAM-1, and IL-27 (Figure 5D).

## 4. Discussion

In the current work, we show that ApTOLL may represent a novel therapeutic target for the treatment of AMI. In pigs subjected to IR, the levels of cardiac troponins [23] were reduced 24 h after administration of ApTOLL, showing a significant improvement of cardiac function by day 7, as a result of left ventricle prevention of necrosis, including preservation of the extracellular matrix, as shown by the reduction of MMP-9 expression. Our results point towards the reduction of the expression of pro-inflammatory cytokines as a mechanism, at least in part, responsible for cardiac protection induced by ApTOLL.

Others have shown that TLR4 correlates with a bad outcome in AMI [24,25]. Several reports show a close correlation between TLR4 and the downstream proinflammatory NF-kappa B transcription factor and ways of inhibition to reduce several inflammatory conditions [9]. However, most results have been gathered in vitro and in murine models of AMI [26], while this is the first report in which, by using a brand-new compound against TLR4, a significant response is achieved in a porcine model of AMI.

Other studies have demonstrated that TLR4 gene silencing reduces the levels of MMP-9 in human aortic smooth muscle cells in the context of atherosclerosis [27]. Our data show that ApTOLL also prevents extracellular matrix degradation, at least by reducing the expression of MMP-9 by day 7 after IR, indicative of cardiovascular injury.

The role of TLR4 in activating the immune response have been thoroughly explored in recent years. TLR4 promotes nuclear NF-kB translocation both in MyD88 dependent and independent pathways and consequently results in the activation of inflammatory pathways and cytokine release [28]. Thus, we evaluated the release of inflammatory-related cytokines at 7 days post-reperfusion, a time point in which the anti-inflammatory-resolution phase is about to start, and, still, TLR4 induced inflammation that was consistently significant [29,30,31,32]. As shown here, ApTOLL significantly reduced the levels of the proinflammatory cytokines implicated in the progression of AMI.

TLR4 increases the levels of IL-1β, IL-6, IL-8, and TNF-α, contributing to inducing cardiac damage in AMI [31,32], while ApTOLL did the opposite, as evidenced in this work. Therefore, ApTOLL may help to maintain heart contractility by preventing cardiac cell death during acute myocardial infarction. In the same way, chemokines that are overexpressed in response to TLR4 and NF-κB [33] were also inhibited, hence improving heart function by preventing the recruitment and migration of immune cells.

Resolving cytokines neutralize and regulate latter stages of the inflammatory response [34]. Two important resolving cytokines, such as IL-4 and IL10, were not affected by ApTOLL, while others in apparent contradiction were downregulated. To fully understand the contribution of ApTOLL in the resolution of inflammation, further investigations should focus on the effect of ApTOLL at later time points to evaluate the contribution of neutrophil and macrophage polarization to a resolving stage [35]. Interestingly, the levels of interleukin 1 receptor antagonist (IL-1RA) were upregulated after treatment with ApTOLL, a cardioprotective molecule that binds to the receptor of IL-1, blocking its activity [36].

The effect of ApTOLL on IL-18 also constitutes a potentially contradictory, since IL-18 is known to induce a pro-inflammatory environment under several circumstances. However, reports showing the pro- and anti-inflammatory properties of IL-18 have emerged, since IL18 binds to IL-18 binding protein (IL-18BP), which mediates IL-37-induced anti-inflammatory effects [37]. Thereby, and besides limitations, including sample size and species of the study, ApTOLL = induced IL18 expression in AMI requires additional investigation.

We used a pig model of coronary ischemia/reperfusion rather than using rodent models, which allows the use of a significant number of individuals at a low economic cost, because the rodent cardiovascular system differs substantially from humans. However, although the procedure used in this work resembles the series of events of patients after reperfusion in the cathlab, the cost of experimentation in these circumstances is rather high when compared to mice, which in the end becomes a strong limitation of the study. Hence, further investigation is required to fully validate our results in patients, as other molecular markers have been tested [38], and to evaluate the relevance of using ApTOLL to prevent the development of the deadly inflammatory response in high-risk populations, as well as to investigate a long-term effect of ApTOLL in heart failure, which represents a significant economic burden given the high number of patients worldwide.

## 5. Conclusions

Blocking the inflammatory response has emerged as one of the main targets to prevent harmful outcomes after AMI. ApTOLL is a promising new tool to achieve this objective, blocking TLR4, one of the upstream molecules that trigger the inflammatory response. Preventing the release of inflammatory cytokines, it is possible to avoid myocardial damage by reducing cardiac fibrosis through prevention of extracellular matrix degradation and, hence, preserving the ventricular function. Further experiments must be performed, but our results make ApTOLL a potential tool for future clinical studies in the onset of AMI.

## 6. Patents

International Application of: APTATARGETS. S.L.

International Application Number: PCT/IB2020/054655

International Filling Date: May 16, 2020

Title: TREATMENT OF TLR-4 MEDIATED DISEASES AND CONDITIONS WITH APTAMERS TARGETING TL4-4.

## Figures and Tables

**Table 1:** Echocardiographic parameters tested by day 3 (D3) and day 7 (D7) after procedure. End diastolic Interventricular septum thickness (IVSD); end diastolic left ventricle internal diameter (LVIDD); end diastolic left ventricular posterior wall thickness (LVPWD); end systolic interventricular septum thickness (IVSS); end systolic left ventricular internal diameter (LVIDS); end systolic left ventricular posterior wall thickness (LVPWS); left ventricle ejection fraction (LVEF); fractional shortening (FS); heart rate (HR); cardiac output (CO). * EF: *p* < 0.001ApTOLL vs. Placebo. FS: *p* < 0.003 ApTOLL vs. Placebo.

PARAMETER	PLACEBO D3	APTOLL D3	PLACEBO D7	APTOLL D7	PARAMETER
**IVSD (mm)**	11.68 ± 0.806	10.857 ± 0.61	10.124 ± 1.357	11.353 ± 1.276	**IVSD (mm)**
**LVIDD (mm)**	37.96 ± 4.219	39.511 ± 4.319	38.488 ± 8.667	38.067 ± 6.052	**LVIDD (mm)**
**LVPWD (mm)**	13.430 ± 2.394	9.954 ± 1.746	10.475 ± 0.67	11.093 ± 2.796	**LVPWD (mm)**
**IVSS (mm)**	15.77 ± 2.264	13.562 ± 0.932	13.153 ± 2.806	14.177 ± 1.678	**IVSS (mm)**
**LVIDS (mm)**	26.28 ± 4.36	29.126 ± 3.493	27.316 ± 5.860	24.705 ± 4.129	**LVIDS (mm)**
**LVPWS (mm)**	15.77 ± 3.383	11.024 ± 1.339	10.926 ± 1.704	11.90 ± 1.722	**LVPWS**
**EF (%)**	49.07 ± 5.91	46.31 ± 2.60	**55.14 ± 4.947**	66.85 ± 3.196 *	**EF (%)**
**FS (%)**	21.25 ± 3.77	20.272 ± 1.443	**28.55 ± 2.530**	**34.75 ± 2.492** *	**FS (%)**
**HR (bpm)**	99 ± 31.494	111.1O1 ± 27.31	103.444 ± 31.269	91.875 ± 11.849	**HR (bpm)**
**CO (mL/sec)**	3614 ± 2068.651	3940.33 ± 1217.115	3801.111 ± 1.826	3918.875 ± 1356	**CO (mL/sec)**

**Table 2:** Plasma levels of cytokines differentially expressed 7 days after procedure.

CYTOKINE	PLACEBO	APTOLL
CCL5	1.108 ± 0.141	0.27 ± 0.070
CXCL1	0.429 ± 0.024	0.105 ± 0.023
CXCL10	1.542 ± 0.237	0.37 ± 0.07
G-CSF	0.372 ± 0.088	0.0732 ± 0.014
GM-CSF	0.261 ± 0.057	0.031 ± 0.009
ICAM-1	0.154 ± 0.030	0.045 ± 0.009
INF-g	0.092 ± 0.019	0
IL1-a	0.255 ± 0.043	0.108 ± 0.018
ILI-b	0.616 ± 0.146	0.047 ± 0.146
IL-IRA	0.193 ± 0.023	0.421 ± 0.123
IL2	0.493 ± 0.074	0.141 ± 0.036
IL5	0.197 ± 0.020	0.014 ± 0.004
IL6	0.309 ± 0.040	0.165 ± 0.043
IL8	0.317 ± 0.042	0.183 ± 0.047
IL12	0.392 ± 0.054	0.213 ± 0.054
IL16	0.306 ± 0.031	0.16 ± 0.046
IL17-A	0.42 ± 0.042	0.109 ± 0.032
IL18	0.311 ± 0.077	0.504 ± 0.116
IL21	0.466 ± 0.074	0.159 ± 0.029
IL27	0.348 ± 0.037	0
MIF	1.359 ± 0.200	0.527 ± 0.089
SERPIN-E1	0.876 ± 0.095	0.454 ± 0.076
TNF-a	0.52 ± 0.040	0.152 ± 0.045
TREM-1	0.4192 ± 0.039	0.105 ± 0.031

## Supplementary Materials

The following are available online at https://www.mdpi.com/2218-273X/10/8/1167/s1, Figure S1: Basal cardiac ultrasound parameters of selected pigs included in the study.
