# Supplementary material for: Targeting TLR4 with ApTOLL Improves Heart Function in Response to Coronary Ischemia Reperfusion in Pigs Undergoing Acute Myocardial Infarction
Source: Biomolecules. 2020 Aug 9;10(8):1167. doi: 10.3390/biom10081167 (PMC7464507; doi:10.3390/biom10081167)
Supplement: Supplementary file 1 [file biomolecules-10-01167-s001.pdf]

biomolecules

*Article*

# Targeting TLR4 with ApTOLL Improves Heart Function in Response to Coronary Ischemia Reperfusion in Pigs Undergoing Acute Myocardial Infarction

**Rafael Ramirez-Carracedo [1,†], Laura Tesoro [1,†], Ignacio Hernandez [1], Javier Diez-Mata [1], David Piñeiro [2], Macarena Hernandez-Jimenez [2], Jose Luis Zamorano [3] and Carlos Zaragoza [1,*]**

**Supplemental Material.**

Basal cardiac ultrasound parameters of selected pigs included in the study.

| PARAMETER | PLACEBO 0 | APTOLL 0 |
|---|---|---|
| IVSD (mm) | 10.324±1.672 | 11.272±1.804 |
| LVIDD (mm) | 37.884±5.134 | 42.237±8.183 |
| LVPWD (mm) | 10.629±1.430 | 11.128±1.537 |
| IVSS (mm) | 13.894±1.887 | 13.991±3.065 |
| LVIDS (mm) | 24.371±3.137 | 26.213±5.726 |
| LVPWS (mm) | 15.954±2.648 | 12.753±1.716 |
| EF (%) | 66.79±3.411 | 68.25±2.571 |
| FS (%) | 36.8±3.425 | 38.2±2.616 |
| HR (bpm) | 93.6±12.971 | 88.8±15.817 |
| CO (ml/sec) | 4089.3±343.218 | 4228.2±470.490 |
